# Sc_3_N@*I*_*h*_-C_80_ as a novel Lewis acid to trap abnormal *N*-heterocyclic carbenes: the unprecedented formation of a singly bonded [6,6,6]-adduct†
†Electronic supplementary information (ESI) available. CCDC 1406974. For ESI and crystallographic data in CIF or other electronic format see DOI: 10.1039/c5sc04070a


**DOI:** 10.1039/c5sc04070a

**Published:** 2015-12-02

**Authors:** Muqing Chen, Lipiao Bao, Min Ai, Wangqiang Shen, Xing Lu

**Affiliations:** a State Key Laboratory of Materials Processing and Die & Mould Technology , School of Materials Science and Engineering , Huazhong University of Science and Technology , 1037 Luoyu Road , Wuhan , 430074 China . Email: lux@hust.edu.cn; b School of Physics and Mechanical & Electronical Engineering , Hubei University of Education , Wuhan 430205 , China

## Abstract

The reaction between an *N*-heterocyclic carbene (NHC), namely 1,3-bis(diisopropylphenyl)-imidazol-2-ylene (**1**), and Sc_3_N@*I*_*h*_-C_80_ successfully affords a Lewis acid–base pair (**2a**). Single crystal X-ray crystallographic results unambiguously reveal the unexpected structure of **2a** where the abnormal carbene center of the NHC is connected to a triple-hexagon-junction (THJ) carbon atom of Sc_3_N@*I*_*h*_-C_80_*via* a single bond. Theoretical calculations reveal that selective entrapment of the abnormal carbene **1** is caused by the steric hindrance between the normal NHC moiety and the fullerene cage, which precludes the formation of normal carbene adducts. Furthermore, the analysis of the electronic density distribution on the cage of Sc_3_N@*I*_*h*_-C_80_ indicates that THJ carbons bear relatively low negative charge densities and, accordingly, are easily attacked by the electron-rich NHC **1** to form the singly bonded [6,6,6]-adduct **2a** instead of the corresponding [5,6,6]-adduct **2b**. It is thus confirmed that the regioselective formation of **2a** is a synergistic effect of both cage size and electron density distribution. Sc_3_N@*I*_*h*_-C_80_, although with a highly charged cage, is proven to show excellent Lewis acidity, opening a wide avenue toward carbon-based Lewis acids taking into account the diversity of endohedral metallofullerenes.

## Abstract 2

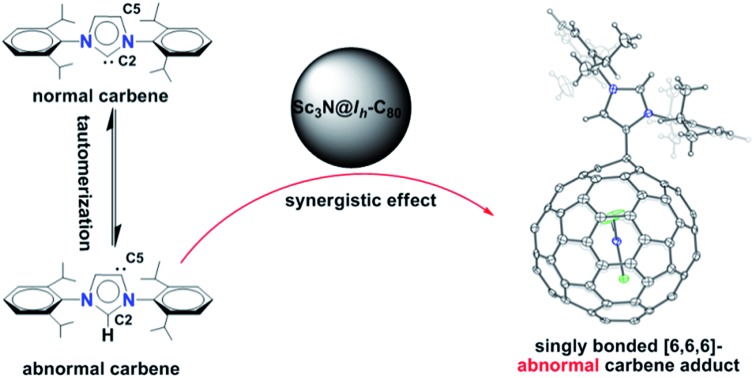
An unprecedented singly bonded [6,6,6]-adduct with an abnormal *N*-heterocyclic carbene structure, represents the first example of carbon-based Lewis acid–base pairs based on endohedral metallofullerenes.

## Introduction

**Scheme 1:**
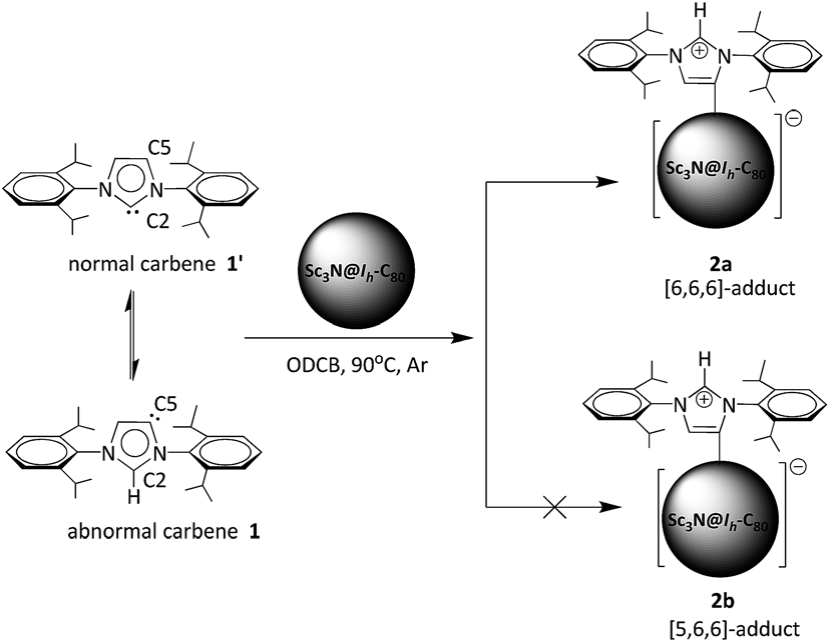
The reaction between **1** and Sc_3_N@*I*_*h*_-C_80_.

“Frustrated Lewis pairs” are promising metal-free catalysts to activate small molecules such as H_2_, CO_2_, alkenes and alkynes.^1^–^3^ Carbon-based Lewis bases are naturally diverse, such as ylides, isonitriles, enamines and *N*-heterocyclic carbenes (NHCs).^4^ Among them, NHCs as stable carbene compounds featuring a neutral divalent carbon atom with two non-bonding electrons are considered as prototypical reactive intermediates and have attracted intensive interest.^5^ Usually, NHCs use the normal carbene center (*e.g.* C2 of **1′** in Scheme 1) to form η_1_ complexes.^6^ However, recent experimental and theoretical results show that abnormal carbenes with C5 as the active center (*e.g.***1** in Scheme 1) have a stronger electron-donating ability and, accordingly, their complexes show better catalytic properties than the normal ones.^7^,^8^ As a direct result, great efforts have been devoted to the exploration of abnormal carbene compounds.^9^

In contrast, carbon-based Lewis acids are limited merely to trityl cations and some electron-poor allenes.^4^ Recently, Bazan and coworkers reported that fullerenes such as C_60_ and C_70_ can behave as all-carbon Lewis acids to form the corresponding Lewis acid–base pairs with a normal NHC structure (**1′**).^10^ This work opens a new perspective on the research of carbon-based Lewis acids. Meanwhile, as a novel class of metal–carbon hybrid molecules, endohedral metallofullerenes (EMFs) feature electron transfer from the internal metallic species to the carbon cage, forming zwitterionic compounds.^11^ Accordingly, it is of special interest to study whether the highly charged carbon cages of EMFs are willing to accept additional electrons to act as Lewis acids or not.

Herein, taking Sc_3_N@*I*_*h*_-C_80_ as a representative, we show that EMFs also exhibit excellent Lewis acidity to form Lewis acid–base pairs with NHCs. Surprisingly, our unambiguous X-ray results reveal that the *abnormal* NHC **1** is bonded to Sc_3_N@*I*_*h*_-C_80_, instead of the normal one **1′** (Scheme 1). More interestingly, a singly bonded [6,6,6]-adduct (**2a**) of Sc_3_N@*I*_*h*_-C_80_ is formed during the reaction, which has never been observed or even predicted, in contrast to the commonly observed [5,6,6]-adduct (**2b**). Our theoretical results reveal that the regioselective formation of the unprecedented [6,6,6]-adduct with an abnormal carbene moiety (**2a**) is synergistically affected by the cage size of Sc_3_N@*I*_*h*_-C_80_ and the electronic density distribution on the cage.

## Results and discussion

**Fig. 1:**
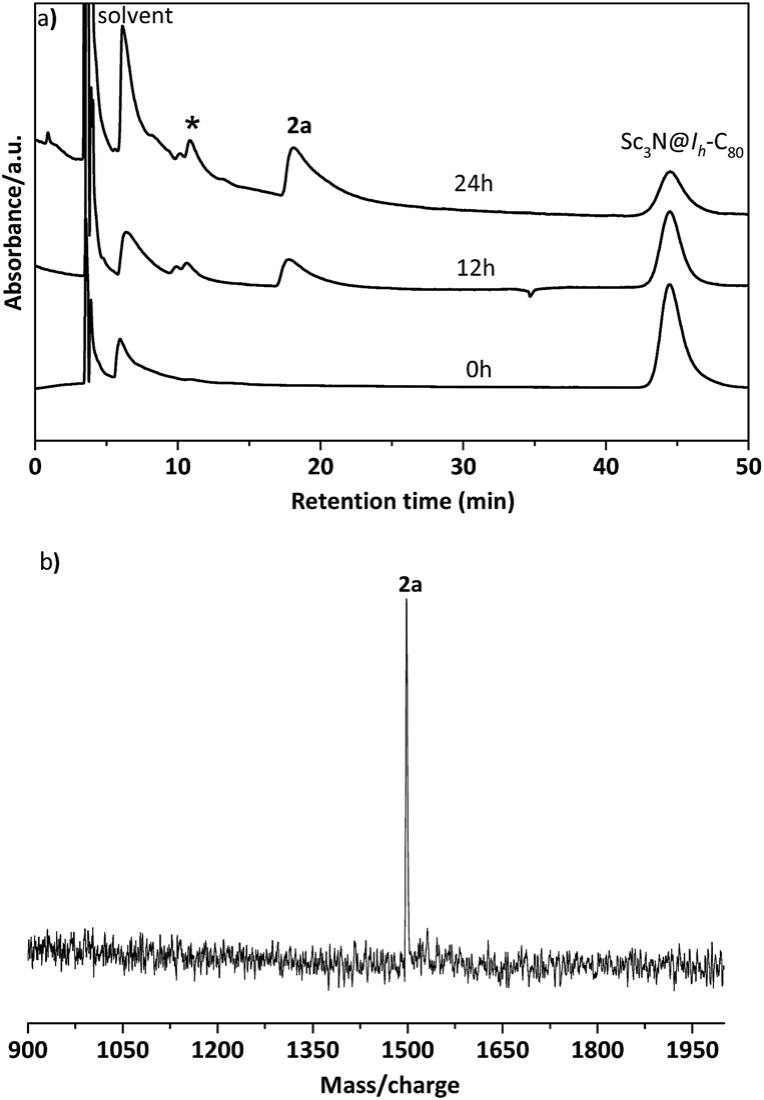
(a) Monitoring the reaction between **1** and Sc_3_N@*I*_*h*_-C_80_*via* HPLC. Conditions: Buckyprep column (*ø* 4.6 mm × 250 mm), 0.8 mL min^–1^ toluene flow, and 330 nm detection wavelength. The peak marked with an asterisk represents an unidentified product. (b) MALDI-TOF mass spectrum of **2a**.

In a typical reaction, an *ortho*-dichlorobenzene (ODCB) solution of Sc_3_N@*I*_*h*_-C_80_ and an excess amount (*ca.* 50-fold) of 1,3-bis(diisopropylphenyl)-imidazol-2-ylene (**1**) was heated to 90 °C under an argon atmosphere (Scheme 1). The reaction progress was monitored *via* high performance liquid chromatography (HPLC). After the solution was heated for 12 hours, a new peak appeared at 18.3 min, which is ascribed to the adduct **2a** as identified using mass spectrometry (Fig. 1). The reaction was terminated after 24 hours, and **2a** was isolated with preparative HPLC in ∼80% conversion yield based on consumed Sc_3_N@*I*_*h*_-C_80_. The matrix assisted laser desorption/ionization time-of-flight (MALDI-TOF) mass spectrum of **2a** displays a single peak at *m*/*z* 1498.2, firmly confirming the successful attachment of the NHC moiety onto the fullerene cage (Fig. 1b).

**Fig. 2:**
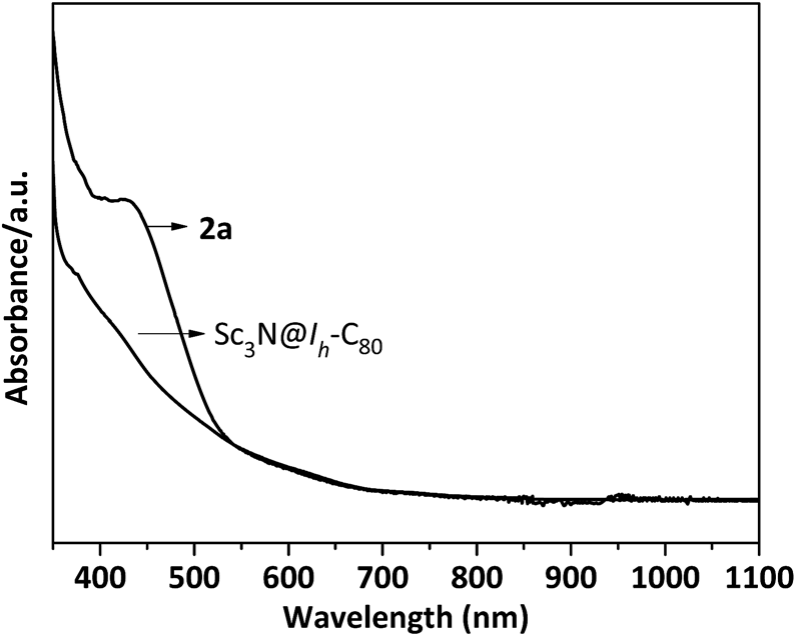
UV-Vis-NIR spectra of Sc_3_N@*I*_*h*_-C_80_ and **2a**.

The electronic configuration of **2a** was investigated using UV-Vis-NIR spectroscopy in toluene (Fig. 2). Although the spectrum of **2a** resembles that of pristine Sc_3_N@*I*_*h*_-C_80_ in the wavelength range between 540 nm and 1100 nm, their curves at 350–540 nm differ significantly from one another, confirming that the electronic structure of Sc_3_N@*I*_*h*_-C_80_ has been altered by the modification.

**Fig. 3:**
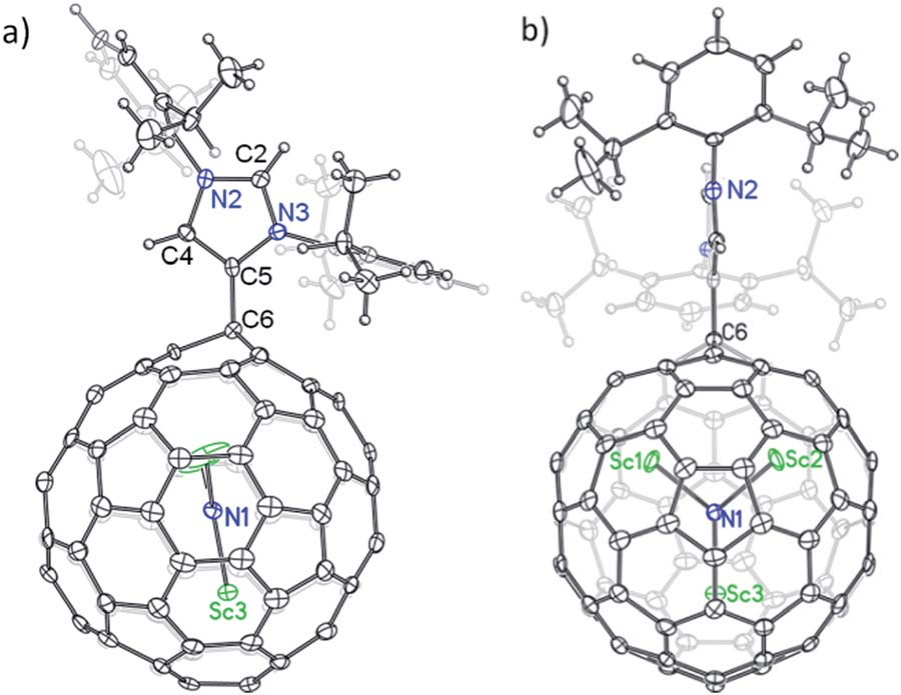
Single-crystal X-ray structure of **2a**: (a) side view, and (b) front view. Thermal ellipsoids are shown at the 50% probability level. Solvent molecules are omitted for clarity.

The structure of **2a** is unequivocally established *via* single-crystal X-ray crystallography. The entire system is fully ordered, including the functionalized cage, the internal cluster and even the three CS_2_ solvent molecules.^12^ It is evident that a single bond is formed between the addend and the cage with a bond length of 1.515 Å (C5–C6), confirming unambiguously the formation of a Lewis acid–base complex (Fig. 3a).^13^,^14^ More surprisingly, the addition site on the cage of Sc_3_N@*I*_*h*_-C_80_ involves a triple hexagon junction (THJ) which is generally less reactive than the carbon atoms of other kinds on a fullerene cage. Such an addition pattern has never been observed or even expected for Sc_3_N@*I*_*h*_-C_80_ because previously reported singly bonded derivatives contained at least two substituents that are exclusively linked to the pentagon–hexagon–hexagon junction (PHHJ) carbon atoms unless the cage is severely functionalized.^15^–^17^ Because of the substitution, the carbon atom at the site of addition (C6) is slightly pulled out from the cage sphere which causes a ‘Y-shaped’ displacement of the internal Sc_3_N cluster with the Sc_3_–N1 bond nearly collinear with the new bond C5–C6, whereas the Sc_3_N-plane is perpendicular to the *N*-heterocyclic ring of the addend (Fig. 3b).

Surprisingly, the NHC moiety is linked to the cage of Sc_3_N@*I*_*h*_-C_80_ with its abnormal carbene center C5 instead of the normal site C2, which is completely different from the corresponding Lewis pairs of C_60_ and C_70_.^10^ The bond length of C4–C5 (1.367 Å) falls into the range of a C=C double bond, confirming the existence of imidazol-2-ylene. The lengths of the other four C–N bonds forming the *N*-heterocyclic ring are similar: 1.384 Å (C4–N2), 1.326 Å (C2–N2), 1.333 Å (C2–N3) and 1.386 Å (C5–N3), excluding the existence of C=N double bonds.

**Fig. 4:**
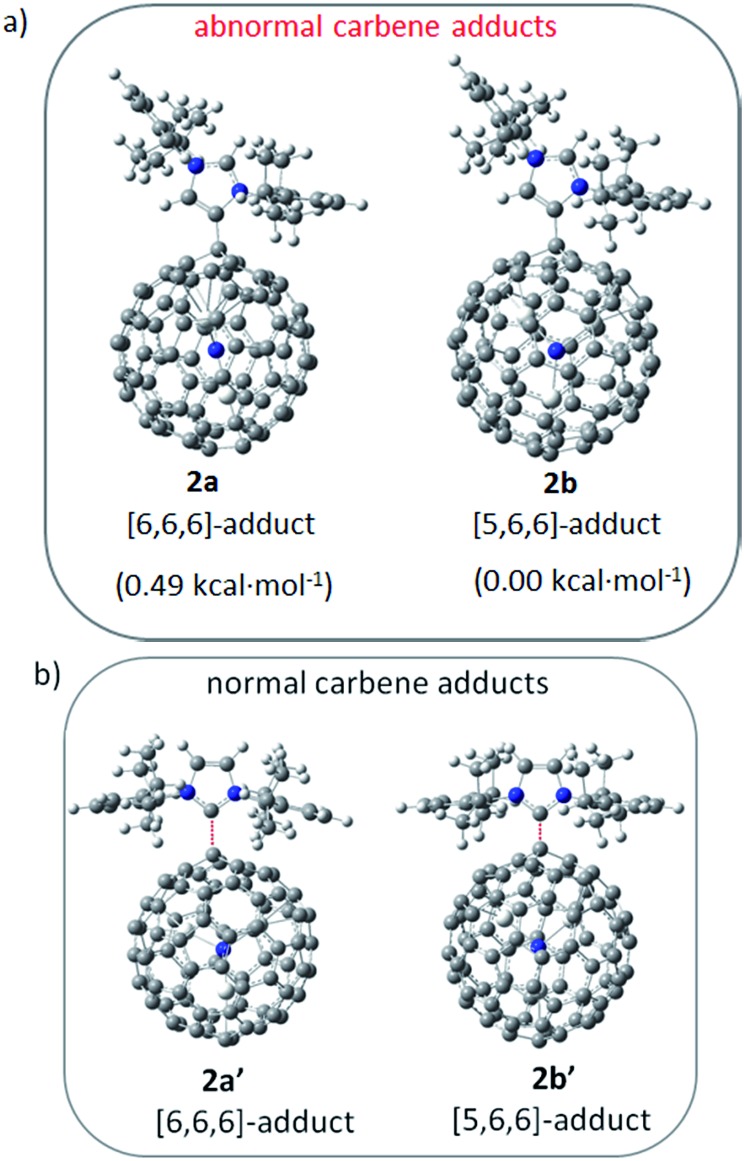
Possible structures of NHC–Sc_3_N@*I*_*h*_-C_80_ complexes and their relative energies calculated at the B3LYP/6-31G*/LANL2DZ (Sc) level.

The unprecedented structure of **2a** with an abnormal NHC moiety singly bonded to a THJ carbon atom is of great interest. First, we try to understand why a [6,6,6]-adduct (**2a**) is formed instead of the corresponding [5,6,6]-adduct (**2b**). It is well-known that THJ carbons are less pyramidal and accordingly are less reactive than the carbon atoms of other kinds of fullerenes.^18^ Indeed, our theoretical results suggest that the [5,6,6]-adduct **2b**, if formed, is 0.49 kcal mol^–1^ more stable than the [6,6,6]-adduct **2a** (Fig. 4a), indicating that the preferential formation of **2a** is not a thermodynamically controlled process. We then consider that the electron density distribution on the cage of Sc_3_N@*I*_*h*_-C_80_ should be a critical factor. It is widely accepted that pentagons are the sites of the negative charges of highly charged fulleride species such as EMFs.^19^ Accordingly, [5,6,6]-junction carbon atoms always accumulate more negative charges than THJ carbons do. As a direct result, NHCs as electron-rich Lewis bases tend to attack the [6,6,6]-junction carbon atoms which have lower electron densities, revealing the Lewis acidic property of Sc_3_N@*I*_*h*_-C_80_. In summary, the [6,6,6]-addition pattern of **2a** is a consequence of the electron density distribution on the cage of Sc_3_N@*I*_*h*_-C_80_.

We then try to find a reasonable explanation for the unexpected formation of the abnormal carbene structure of **2a**. Usually NHCs use the normal carbene center (C2 in **1′**, Scheme 1) to bind metals. However, abnormal NHC carbene species (**1**, Scheme 1) can stably exist and can even be isolated.^20^ Several examples of abnormal carbene complexes have been reported, which show enhanced catalytic properties for the activation of unreactive bonds.^7^,^8^ Furthermore, Dagorne and coworkers reported that a normal but sterically congested NHC–AlMe_3_ Lewis acid–base pair can isomerize to its abnormal NHC–AlMe_3_ species.^20^ This result inspires us to speculate that the abnormal carbene structure of **2a** is also caused by a steric effect. Our computational results showed that neither the normal [6,6,6]-adduct (**2a′**) nor the normal [5,6,6]-adduct (**2b′**) can exist as stable compounds: during the optimization processes the single bond connecting the normal NHC moiety and Sc_3_N@*I*_*h*_-C_80_ is broken (Fig. 4b), which can be attributed to the steric hindrance between the congested diisopropylphenyl groups of the normal NHC (**1′**) and the large cage of Sc_3_N@*I*_*h*_-C_80_.

**Scheme 2:**
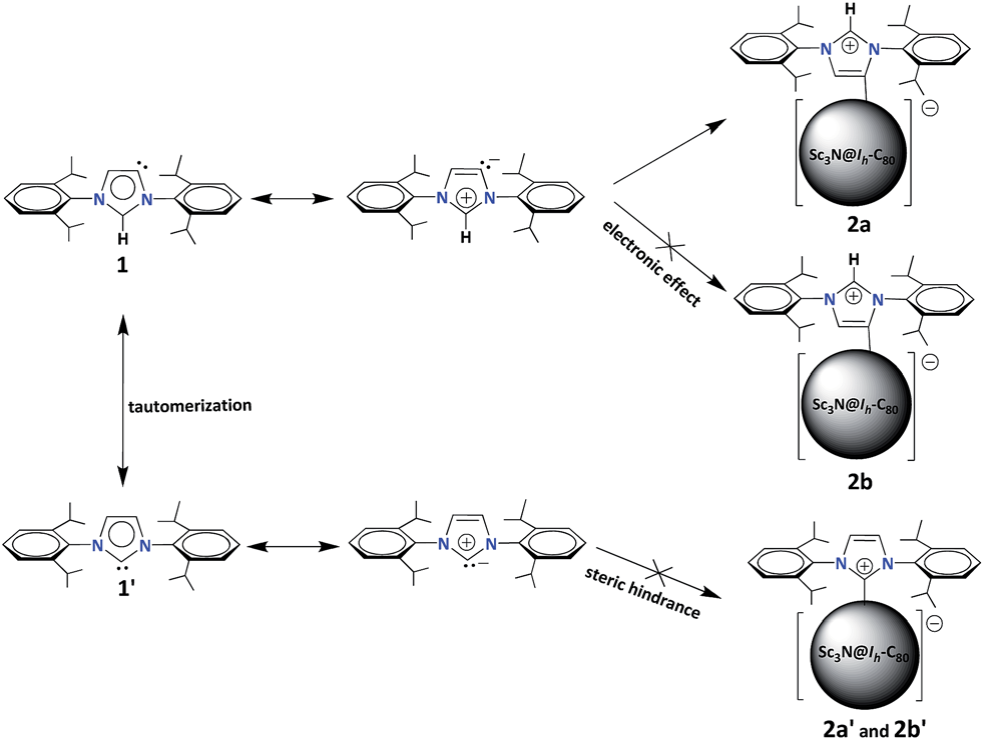
Plausible formation mechanism of **2a**.

Finally, we propose a plausible mechanism to rationalize the unexpected formation of **2a** (Scheme 2). According to the literature, the normal carbene **1′**, where C2 is the carbene center, can tautomerize into the abnormal one **1** with C5 as the active site.^21^ Then, the tautomers (**1** and **1′**) turn into the corresponding mesoionic compounds.^9^ Since the mesoionic species of **1′** can not form stable adducts (**2a′**and **2b′**) with Sc_3_N@*I*_*h*_-C_80_ because of the high steric hindrance between the addend and the cage, only the abnormal carbene structure is possible. As discussed above, the electron-rich NHC **1** (or the corresponding mesoionic compound) tends to attack one of the THJ carbon atoms with low electron densities, forming the [6,6,6]-adduct **2a** in a highly regioselective manner.

## Conclusions

In summary, Sc_3_N@*I*_*h*_-C_80_ is confirmed to be an excellent carbon-based Lewis acid although its cage is negatively charged, representing the first example of EMFs that readily undergo Lewis acid–base complexation reactions with NHCs. The regioselective formation of the unusual singly bonded [6,6,6]-adduct **2a** is reasonably interpreted by analyzing the charge density distribution on the cage because the electron-rich NHC is prone to attack one of the THJ carbons with low electron densities. More interestingly, Sc_3_N@*I*_*h*_-C_80_ here is found to selectively trap the rare abnormal NHC **1** as a consequence of the steric hindrance between the normal NHC moiety and Sc_3_N@*I*_*h*_-C_80_. Hence, we conclude that the regioselective and unprecedented formation of **2a** is a synergistic effect of both the cage size and electron density distribution of Sc_3_N@*I*_*h*_-C_80_. This synthetic strategy can be easily extended to create various EMF-based Lewis acid–base pairs with different metallic cores and/or cage structures, which may show unique catalytic properties in organic synthesis, taking into account their “frustrated” characteristics.

## Supplementary Material

Supplementary information

Crystal structure data
