# Supplementary material for: Sc3N@Ih-C80 as a novel Lewis acid to trap abnormal N-heterocyclic carbenes: the unprecedented formation of a singly bonded [6,6,6]-adduct
Source: Chem Sci. 2015 Dec 2;7(3):2331–4. doi: 10.1039/c5sc04070a (PMC6003601; doi:10.1039/c5sc04070a)
Supplement: Supplementary file 1 [file SC-007-C5SC04070A-s001.pdf]

Electronic Supplementary Material (ESI) for Chemical Science.
This journal is © The Royal Society of Chemistry 2015

**Supporting Information of**

# $Sc_3N@I_h-C_{80}$ as a Novel Lewis Acid to Trap Abnormal N-Heterocyclic Carbene: Unprecedented Formation of a Singly Bonded [6,6,6]-adduct

Muqing Chen,[a,b] Lipiao Bao,[a] Min Ai,[a] Wangqiang Shen,[a] Xing Lu*,[a]

[a]State Key Laboratory of Materials Processing and Die & Mould Technology, School of Materials Science and Engineering, Huazhong University of Science and Technology, 1037 Luoyu Road, Wuhan 430074, China.

[b]School of Physics and Mechanical Engineering, Hubei University of Education, Wuhan 430205, China.

## Experimental

**General methods:** High-performance liquid chromatography (HPLC) was conducted on an LC-9130 NEXT machine (Japan Analytical Industry Co., Ltd.) with toluene as mobile phase. Matrix-assisted laser desorption/ionization time-of-flight (MALDI-TOF) mass spectrometry was measured on a BIFLEX III spectrometer (Bruker Daltonics Inc., Germany) using 1,1,4,4-tetraphenyl-1, 3-butadiene as matrix. UV-Vis-NIR spectra were obtained from a PE Lambda 750S spectrometer in toluene.

**Synthesis of 2a**: $Sc_3N@I_h-C_{80}$ was synthesized with a direct-current arc discharge method and was isolated with HPLC. A mixture of $Sc_3N@I_h-C_{80}$ (~5 mg, 4.5 μmol) and 1,3-bis(diisopropyl- phenyl)imidazol-2-ylene (87.5 mg, 0.22 mmol) dissolved in 10 mL *ortho*-dichlorobenzene (ODCB) was stirred at 90 °C in dark under argon atmosphere for 24 h. After removal of ODCB, the residual solid was dissolved in toluene and was subjected to HPLC separation.

**Single crystal XRD measurement of 2a.** A piece of crystal with dimensions of 0.04 × 0.02 × 0.02 mm was chosen for the single-crystal XRD measurement and provided all the structural information about **2a**. X-ray data were collected at 90 K on a Bruker Apex II machine equipped with a CCD detector (Bruker AXS Inc., Germany). The multi-scan method was used for absorption corrections. The structure was solved by using direct method and was refined with SHELXL-2014.[S1] CCDC-1406974 contains the supplementary crystallographic data for this paper which can be obtained free of charge from the Cambridge Crystallographic Data Centre via www.ccdc.cam.ac.uk/ data_request/cif.

**Theoretical Calculations.** DFT calculations were conducted with Gaussian 09 package[S2] using the B3LYP functional with the 6-31G basis set[S3] for C, H, N atoms and the ~dz basis set[S4] (with the ~dz effective core potential) for Sc atoms.

**REFERENCES**

S1. *G. M. Sheldrick, Acta Crystallogr., Sect. A: Found. Crystallogr.,* 2008, ***64***, 112.

S2. M. J. Frisch, G. W. Trucks, H. B. Schlegel, G. E. Scuseria, M. A. Robb, J. R. Cheeseman, G. Scalmani, V. Barone, B. Mennucci, G. A. Petersson, H. Nakatsuji, M. Caricato, X. Li, H. P. Hratchian, A. F. Izmaylov, J. Bloino, G. Zheng, J. L. Sonnenberg, M. Hada, M. Ehara, K. Toyota, R. Fukuda, J. Hasegawa, M. Ishida, T. Nakajima, Y. Honda, O. Kitao, H. Nakai, T. Vreven, J. A. Montgomery Jr., J. E. Peralta, F. Ogliaro, M. J. Bearpark, J. Heyd, E. N. Brothers, K. N. Kudin, V. N. Staroverov, R. Kobayashi, J. Normand, K. Raghavachari, A. P. Rendell, J. C. Burant, S. S. Iyengar, J. Tomasi, M. Cossi, N. Rega, N. J. Millam, M. Klene, J. E. Knox, J. B. Cross, V. Bakken, C. Adamo, J. Jaramillo, R. Gomperts, R. E. Stratmann, O. Yazyev, A. J. Austin, R. Cammi, C. Pomelli, J. W. Ochterski, R. L. Martin, K. Morokuma, V. G. Zakrzewski, G. A. Voth, P. Salvador, J. J. Dannenberg, S. Dapprich, A. D. Daniels, Ö. Farkas, J. B. Foresman, J. V. Ortiz, J. Cioslowski, D. J. Fox, Gaussian 09; revision A.02; Gaussian, Inc.: Wallingford, CT, USA, 2009.

S3. W. J. Hehre, R. Ditchfield, J. A. Pople, *J. Chem. Phys.* 1972, ***56***, 2257.

S4. P. J. Hay, W. R. Wadt, *J. Chem. Phys.* 1985, ***82***, 299.

**Table S1**. Cartesian coordinates and energies of **2a** and **2b** optimized at the B3LYP/6-31G*/LANL2DZ(Sc) level.

**2a**

xyz file:

| | | | |
|---|---|---|---|
| C | -1.2098 | 7.2807 | 4.1542 |
| C | -1.3202 | 8.3907 | 3.264 |
| C | -0.2156 | 8.9692 | 2.5716 |
| C | 1.0608 | 8.4144 | 2.8958 |
| C | 1.2484 | 7.3156 | 3.7878 |
| C | 0.0765 | 6.4808 | 4.3227 |
| C | 0.3137 | 6.4837 | 5.8118 |
| C | -0.7245 | 6.5764 | 6.7917 |
| C | -2.0605 | 6.9854 | 6.4914 |
| C | -2.2634 | 7.35 | 5.1158 |
| C | -3.0445 | 8.5658 | 4.7971 |
| C | -2.4458 | 9.1749 | 3.6578 |
| C | -2.4853 | 10.5894 | 3.4325 |
| C | -1.415 | 11.1508 | 2.7053 |
| C | -0.2771 | 10.3655 | 2.2914 |
| C | 0.8993 | 11.1849 | 2.3482 |
| C | 2.143 | 10.6461 | 2.7108 |
| C | 2.2443 | 9.2375 | 2.9453 |
| C | 3.1604 | 8.6418 | 3.8556 |
| C | 2.5453 | 7.42 | 4.3886 |
| C | 2.7673 | 7.0393 | 5.7679 |
| C | 1.589 | 6.5953 | 6.4346 |
| C | 1.3458 | 6.8045 | 7.8394 |
| C | -0.0604 | 6.7992 | 8.0391 |
| C | -0.6627 | 7.5892 | 9.0447 |
| C | -1.9819 | 8.0647 | 8.7644 |
| C | -2.6817 | 7.7648 | 7.5409 |
| C | -3.4666 | 8.958 | 7.213 |
| C | -3.6092 | 9.3808 | 5.8393 |
| C | -3.6576 | 10.7994 | 5.5993 |
| C | -3.1348 | 11.38 | 4.3996 |
| C | -2.6622 | 12.6893 | 4.7147 |
| C | -1.5257 | 13.2235 | 4.0553 |
| C | -0.9235 | 12.4757 | 3.0186 |
| C | 0.5024 | 12.4893 | 2.7951 |
| C | 1.3646 | 13.2585 | 3.6102 |
| C | 2.672 | 12.7669 | 3.8941 |
| C | 3.0605 | 11.4539 | 3.4417 |
| C | 3.9531 | 10.8843 | 4.4271 |

| | | | |
|---|---|---|---|
| C | 3.9992 | 9.4833 | 4.6762 |
| C | 4.2719 | 9.0516 | 6.0096 |
| C | 3.6691 | 7.8478 | 6.5683 |
| C | 3.3521 | 8.1278 | 7.9475 |
| C | 2.2056 | 7.6102 | 8.6197 |
| C | 1.6108 | 8.3592 | 9.6821 |
| C | 0.1903 | 8.3484 | 9.8836 |
| C | -0.212 | 9.651 | 10.3433 |
| C | -1.4633 | 10.1974 | 9.977 |
| C | -2.3547 | 9.3944 | 9.2168 |
| C | -3.2552 | 9.9388 | 8.2497 |
| C | -3.2664 | 11.3404 | 7.9805 |
| C | -3.513 | 11.7457 | 6.6635 |
| C | -2.8909 | 12.9259 | 6.0994 |
| C | -1.997 | 13.6967 | 6.8906 |
| C | -0.895 | 14.3621 | 6.2239 |
| C | -0.6476 | 14.0511 | 4.8594 |
| C | 0.7498 | 14.0726 | 4.6377 |
| C | 1.3912 | 14.3813 | 5.8777 |
| C | 2.6584 | 13.7543 | 6.1781 |
| C | 3.3069 | 13.0024 | 5.1561 |
| C | 4.0883 | 11.8553 | 5.4931 |
| C | 4.2543 | 11.4225 | 6.8247 |
| C | 4.3501 | 10.0305 | 7.0775 |
| C | 3.8142 | 9.4618 | 8.2644 |
| C | 3.1789 | 10.2507 | 9.2736 |
| C | 2.0892 | 9.6798 | 9.999 |
| C | 0.9715 | 10.4538 | 10.4294 |
| C | 0.9026 | 11.8487 | 10.118 |
| C | -0.3697 | 12.4172 | 9.8469 |
| C | -1.5416 | 11.61 | 9.7828 |
| C | -2.4205 | 12.158 | 8.7846 |
| C | -1.794 | 13.3029 | 8.2204 |
| C | -0.5254 | 13.4765 | 8.8798 |
| C | 0.5895 | 14.089 | 8.2223 |
| C | 0.3749 | 14.6083 | 6.8815 |
| C | 1.858 | 13.4993 | 8.5208 |
| C | 2.8907 | 13.3646 | 7.5171 |
| C | 3.6882 | 12.2281 | 7.8523 |
| C | 3.1601 | 11.6629 | 9.0447 |
| C | 2.0298 | 12.4385 | 9.4696 |
| C | 0.0277 | 5.0519 | 3.8226 |
| C | 0.0742 | 3.9056 | 4.5663 |
| H | 0.1271 | 3.8515 | 5.5133 |

| | | | |
|---|---|---|---|
| C | -0.0584 | 3.3028 | 2.4489 |
| H | -0.1181 | 2.7802 | 1.6576 |
| C | 0.0706 | 1.4346 | 4.0626 |
| C | 1.3182 | 0.8682 | 4.2897 |
| C | 1.3124 | -0.4545 | 4.7275 |
| H | 2.1396 | -0.8804 | 4.9198 |
| C | 0.1505 | -1.1629 | 4.8923 |
| H | 0.1837 | -2.0621 | 5.1945 |
| C | -1.078 | -0.5715 | 4.6176 |
| H | -1.8771 | -1.0753 | 4.7165 |
| C | -1.1428 | 0.7567 | 4.1963 |
| C | 2.6221 | 1.6084 | 4.0406 |
| H | 2.3872 | 2.5005 | 3.656 |
| C | 3.4989 | 0.8848 | 3.0076 |
| H | 2.9856 | 0.7399 | 2.1852 |
| H | 3.7862 | 0.0207 | 3.3702 |
| H | 4.2866 | 1.4318 | 2.8079 |
| C | 3.402 | 1.8558 | 5.2843 |
| H | 4.1783 | 2.4166 | 5.0773 |
| H | 3.706 | 1.0009 | 5.6561 |
| H | 2.8342 | 2.3138 | 5.94 |
| C | -2.4965 | 1.39 | 3.9216 |
| H | -2.3421 | 2.3386 | 3.6468 |
| C | -3.3814 | 1.3991 | 5.1597 |
| H | -4.2648 | 1.754 | 4.9271 |
| H | -2.9738 | 1.9637 | 5.8484 |
| H | -3.4767 | 0.4841 | 5.5004 |
| C | -3.1952 | 0.6738 | 2.7658 |
| H | -4.0535 | 1.1083 | 2.5845 |
| H | -3.3486 | -0.2623 | 3.0076 |
| H | -2.6312 | 0.7172 | 1.9654 |
| C | -0.0851 | 5.4878 | 1.306 |
| C | -1.3409 | 5.8989 | 0.8535 |
| C | -1.3516 | 6.7129 | -0.2747 |
| H | -2.1783 | 7.0238 | -0.6264 |
| C | -0.1566 | 7.0755 | -0.8902 |
| H | -0.1822 | 7.6262 | -1.6631 |
| C | 1.0692 | 6.6503 | -0.3993 |
| H | 1.8696 | 6.9233 | -0.8316 |
| C | 1.1335 | 5.8315 | 0.7162 |
| C | -2.6243 | 5.4454 | 1.5184 |
| H | -2.4169 | 5.235 | 2.4727 |
| C | -3.1671 | 4.1542 | 0.8554 |
| H | -3.3924 | 4.336 | -0.0806 |

| | | | |
|---|---|---|---|
| H | -3.9697 | 3.8555 | 1.3316 |
| H | -2.4834 | 3.4531 | 0.8975 |
| C | -3.7155 | 6.515 | 1.5019 |
| H | -4.0578 | 6.6197 | 0.5898 |
| H | -3.3423 | 7.3679 | 1.8115 |
| H | -4.4465 | 6.2457 | 2.0954 |
| C | 2.4479 | 5.2724 | 1.2364 |
| H | 2.3276 | 5.0421 | 2.2016 |
| C | 3.5758 | 6.2836 | 1.1283 |
| H | 3.7863 | 6.4395 | 0.1832 |
| H | 4.3701 | 5.9364 | 1.5862 |
| H | 3.3 | 7.1266 | 1.5459 |
| C | 2.8219 | 3.9877 | 0.4836 |
| H | 2.9629 | 4.1949 | -0.4652 |
| H | 2.0959 | 3.336 | 0.5715 |
| H | 3.6451 | 3.6155 | 0.8627 |
| N | 0.3343 | 10.3777 | 6.2642 |
| N | 0.0312 | 2.8373 | 3.6871 |
| N | -0.05 | 4.6343 | 2.5039 |
| Sc | 1.8618 | 9.1281 | 5.8483 |
| Sc | -1.2147 | 9.0861 | 6.2126 |
| Sc | 0.3428 | 12.358 | 6.6163 |

E= -4402.81785765 a.u.

**2b**

xyz file:

| | | | |
|---|---|---|---|
| C | -0.99167 | 1.67628 | -1.48097 |
| C | -0.02352 | 1.87101 | -2.53143 |
| C | 0.41098 | 0.80207 | -3.37509 |
| C | -0.16921 | -0.48244 | -3.13507 |
| C | -1.14175 | -0.7051 | -2.09216 |
| C | -1.49103 | 0.36563 | -1.20925 |
| C | -2.18036 | 0.06707 | 0.10744 |
| C | -1.56291 | 1.00094 | 1.18265 |
| C | -1.00872 | 2.28232 | 0.91636 |
| C | -0.81257 | 2.71097 | -0.47896 |
| C | 0.36853 | 3.51024 | -0.89255 |
| C | 0.83244 | 2.96059 | -2.16252 |
| C | 2.21334 | 2.95152 | -2.59003 |
| C | 2.62706 | 1.9122 | -3.47933 |
| C | 1.7291 | 0.85431 | -3.88385 |
| C | 2.49308 | -0.35969 | -4.05691 |
| C | 1.94121 | -1.64328 | -3.75658 |
| C | 0.55626 | -1.70126 | -3.34591 |
| C | 0.00133 | -2.73403 | -2.47847 |

| | | | |
|---|---|---|---|
| C | -1.10329 | -2.1072 | -1.70644 |
| C | -1.28046 | -2.37934 | -0.26814 |
| C | -1.6538 | -1.30832 | 0.58727 |
| C | -1.24131 | -1.19283 | 1.95868 |
| C | -1.17388 | 0.19683 | 2.31535 |
| C | -0.17191 | 0.6582 | 3.23072 |
| C | 0.36788 | 1.96575 | 3.00462 |
| C | -0.02314 | 2.75937 | 1.8591 |
| C | 1.12815 | 3.55627 | 1.45754 |
| C | 1.3783 | 3.90377 | 0.07503 |
| C | 2.75316 | 3.91518 | -0.34651 |
| C | 3.16427 | 3.48869 | -1.67499 |
| C | 4.48024 | 2.92089 | -1.57657 |
| C | 4.8598 | 1.81527 | -2.39762 |
| C | 3.94816 | 1.34606 | -3.38564 |
| C | 3.86573 | -0.05519 | -3.74191 |
| C | 4.69147 | -1.03421 | -3.12054 |
| C | 4.16873 | -2.34677 | -2.9127 |
| C | 2.80164 | -2.65074 | -3.23208 |
| C | 2.3103 | -3.6098 | -2.25621 |
| C | 0.93459 | -3.64809 | -1.84148 |
| C | 0.68551 | -3.98256 | -0.45505 |
| C | -0.38293 | -3.35378 | 0.3092 |
| C | 0.06301 | -3.24163 | 1.68224 |
| C | -0.33041 | -2.145 | 2.51755 |
| C | 0.58306 | -1.70065 | 3.51146 |
| C | 0.66359 | -0.30396 | 3.86439 |
| C | 2.03831 | -0.00413 | 4.18216 |
| C | 2.60251 | 1.26912 | 3.87294 |
| C | 1.73557 | 2.26797 | 3.31787 |
| C | 2.21249 | 3.23327 | 2.36092 |
| C | 3.57297 | 3.23199 | 1.92294 |
| C | 3.82919 | 3.60788 | 0.56514 |
| C | 4.89436 | 2.99854 | -0.19277 |
| C | 5.71687 | 1.98852 | 0.39512 |
| C | 6.2312 | 0.93446 | -0.45421 |
| C | 5.73555 | 0.83536 | -1.8088 |
| C | 5.65439 | -0.5552 | -2.16227 |
| C | 6.09902 | -1.34048 | -1.03223 |
| C | 5.44731 | -2.60706 | -0.77203 |
| C | 4.53478 | -3.11936 | -1.74586 |
| C | 3.38856 | -3.89383 | -1.3392 |
| C | 3.13846 | -4.18458 | 0.04003 |
| C | 1.77428 | -4.24612 | 0.46257 |

| | | | |
|---|---|---|---|
| C | 1.38023 | -3.8035 | 1.77569 |
| C | 2.33551 | -3.29066 | 2.71508 |
| C | 1.90671 | -2.26449 | 3.60895 |
| C | 2.80373 | -1.21816 | 4.03355 |
| C | 4.1482 | -1.1764 | 3.55865 |
| C | 4.7375 | 0.10747 | 3.33496 |
| C | 3.97832 | 1.31876 | 3.49484 |
| C | 4.45407 | 2.28896 | 2.53593 |
| C | 5.50199 | 1.67166 | 1.77065 |
| C | 5.68159 | 0.31528 | 2.26099 |
| C | 6.14553 | -0.75076 | 1.42076 |
| C | 6.48999 | -0.42071 | 0.04075 |
| C | 5.54312 | -2.02915 | 1.6649 |
| C | 5.2302 | -2.96086 | 0.59411 |
| C | 4.10009 | -3.74862 | 1.00259 |
| C | 3.70727 | -3.3053 | 2.32049 |
| C | 4.59883 | -2.25543 | 2.73492 |
| C | -3.58084 | 0.02977 | 0.08112 |
| C | -4.3492 | -1.09653 | 0.22019 |
| H | -4.04973 | -2.11173 | 0.38459 |
| C | -5.74996 | 0.59341 | -0.0879 |
| H | -6.64703 | 1.16772 | -0.20562 |
| C | -6.82345 | -1.64464 | 0.20736 |
| C | -7.2659 | -2.28489 | -0.96875 |
| C | -8.34262 | -3.1736 | -0.84329 |
| H | -8.71119 | -3.68603 | -1.72043 |
| C | -8.9401 | -3.41173 | 0.39219 |
| H | -9.76781 | -4.10406 | 0.46484 |
| C | -8.47284 | -2.76842 | 1.53595 |
| H | -8.94053 | -2.96993 | 2.48888 |
| C | -7.40037 | -1.86815 | 1.47477 |
| C | -6.60895 | -2.07058 | -2.33226 |
| H | -5.86099 | -1.28118 | -2.23499 |
| C | -7.62591 | -1.60377 | -3.39707 |
| H | -8.14983 | -0.69985 | -3.0817 |
| H | -8.37436 | -2.37142 | -3.60087 |
| H | -7.11157 | -1.38874 | -4.33526 |
| C | -5.86363 | -3.3434 | -2.79733 |
| H | -5.35997 | -3.15873 | -3.74751 |
| H | -6.55535 | -4.17607 | -2.9389 |
| H | -5.10915 | -3.65262 | -2.07278 |
| C | -6.88451 | -1.20762 | 2.7532 |
| H | -6.14566 | -0.45246 | 2.47803 |
| C | -6.16456 | -2.23637 | 3.65708 |

| | | | |
|---|---|---|---|
| H | -5.75877 | -1.7423 | 4.5414 |
| H | -5.33817 | -2.72025 | 3.13468 |
| H | -6.85246 | -3.01523 | 3.99201 |
| C | -8.00722 | -0.48231 | 3.5269 |
| H | -7.58786 | 0.04245 | 4.38689 |
| H | -8.75464 | -1.18327 | 3.90256 |
| H | -8.51867 | 0.2491 | 2.89883 |
| C | -4.23207 | 2.51969 | -0.3084 |
| C | -4.16176 | 3.34607 | 0.83281 |
| C | -3.93148 | 4.71049 | 0.61482 |
| H | -3.85374 | 5.37669 | 1.46103 |
| C | -3.79697 | 5.2214 | -0.6737 |
| H | -3.60907 | 6.27689 | -0.81603 |
| C | -3.90714 | 4.38344 | -1.77989 |
| H | -3.81016 | 4.79802 | -2.77221 |
| C | -4.13272 | 3.00918 | -1.62769 |
| C | -4.39895 | 2.8195 | 2.24956 |
| H | -4.20183 | 1.74515 | 2.25146 |
| C | -5.87704 | 3.03653 | 2.66075 |
| H | -6.11472 | 4.10185 | 2.68747 |
| H | -6.05749 | 2.62552 | 3.65591 |
| H | -6.57351 | 2.56073 | 1.9667 |
| C | -3.46198 | 3.44292 | 3.30366 |
| H | -3.67817 | 4.50102 | 3.46306 |
| H | -2.41699 | 3.34592 | 3.01744 |
| H | -3.59788 | 2.93589 | 4.26034 |
| C | -4.32926 | 2.11892 | -2.85676 |
| H | -4.13654 | 1.0844 | -2.56194 |
| C | -3.35833 | 2.44213 | -4.01057 |
| H | -3.5708 | 3.41483 | -4.45811 |
| H | -3.46019 | 1.69288 | -4.79751 |
| H | -2.32423 | 2.43939 | -3.67347 |
| C | -5.79192 | 2.20879 | -3.35982 |
| H | -6.0234 | 3.22621 | -3.68121 |
| H | -6.51359 | 1.93581 | -2.58715 |
| H | -5.94138 | 1.54308 | -4.21199 |
| N | 2.25227 | -0.11014 | -0.2284 |
| N | -5.69006 | -0.73383 | 0.11364 |
| N | -4.49586 | 1.09206 | -0.11572 |
| Sc | 0.86341 | -1.47396 | -0.75967 |
| Sc | 1.05462 | 1.50335 | -0.0211 |
| Sc | 4.20213 | -0.28536 | 0.02442 |

E= -4402.81707388 a.u.
